# Saul Hertz, MD, and the birth of radionuclide therapy

**DOI:** 10.1186/s40658-017-0182-7

**Published:** 2017-04-27

**Authors:** Frederic H. Fahey, Frederick D. Grant, James H. Thrall

**Affiliations:** 1 Department of Radiology, Harvard Medical School, Boston, MA USA; 2 Department of Radiology, Boston Children’s Hospital, Boston, MA USA; 3 Department of Radiology, Mount Auburn Hospital, Boston, MA USA; 4 Department of Radiology, Massachusetts General Hospital, Boston, MA USA

**Keywords:** History, Saul Hertz, Radioiodine therapy

## Abstract

The year, 2016, marked the 75th anniversary of Dr. Saul Hertz first using radioiodine to treat a patient with thyroid disease. In November of 1936, a luncheon was held of the faculty of Harvard Medical School where Karl Compton, PhD, president of the Massachusetts Institute of Technology was invited to give a presentation entitled “What Physics Can Do for Biology and Medicine.” Saul Hertz who attended the luncheon spontaneously asked the very pertinent question that perhaps changed the course of treatment of thyroid disease, “Could iodine be made radioactive artificially?” We review the events leading up to the asking of this question, the preclinical investigations by Dr. Hertz and his colleague Arthur Roberts prior to the treatment of the first patient and what occurred in the years following this landmark event. This commentary seeks to set the record straight to the sequence of events leading to the first radioiodine therapy, so that those involved can be recognized with due credit.

## Background

The year, 2016, marked the 75th anniversary of Dr. Saul Hertz first using radioiodine to treat a patient with thyroid disease. In honor of this anniversary, we have spent a bit of time researching this wonderful advance, including what led up and was subsequent to this event. The excellent and informative history of nuclear medicine presented in 2014 by Peter Ell [1] describes a conversation between Howard Means of the Massachusetts General Hospital (MGH) and Robley Evans of the Massachusetts Institute of Technology (MIT) that most likely did not occur. This misunderstanding is not surprising, given that many versions of these events have been presented and published over the years. In this commentary, we hope to set the record straight to the sequence of events leading to the first radioiodine therapy, so that those involved can be recognized with due credit.

On November 12, 1936, Karl Compton, president of MIT, was invited to give a presentation at a luncheon of the faculty of Harvard Medical School. His topic was “What Physics Can Do for Biology and Medicine.” Karl Compton was a physicist and the older brother of Arthur Compton, noted for characterizing the photon interaction that came to be known as “Compton scattering.” Dr. Compton had been appointed president of MIT in 1930. It is likely that, during his presentation, Compton touched upon the recent production of artificial radioactivity by Frederic and Iréne Joliot Curie [2].

**Fig. 1:**
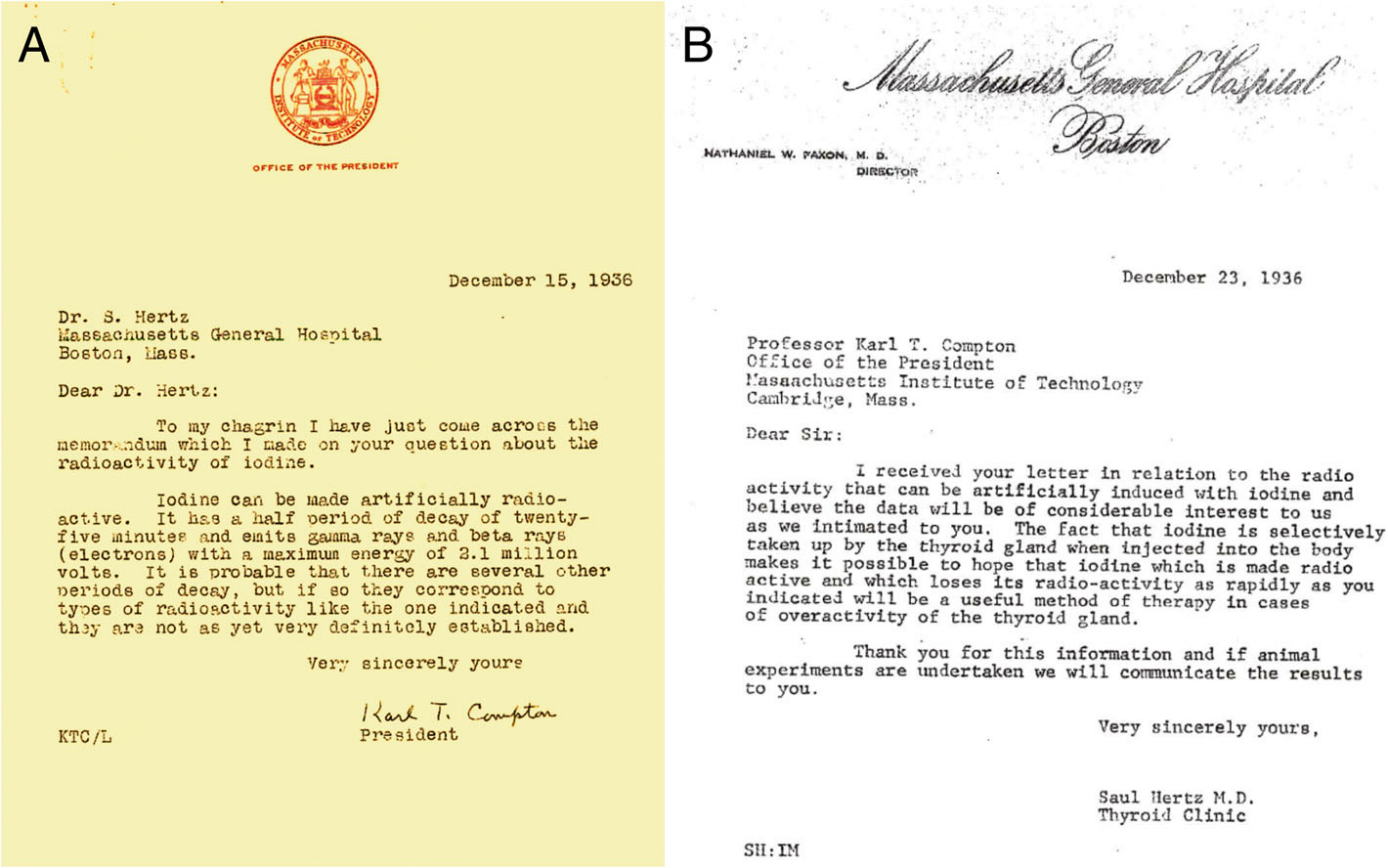
**a** Letter from Karl Compton, president of the Massachusetts Institute of Technology, to Dr. Saul Hertz dated December 15, 1936. In the letter, Dr. Compton clearly indicates that the question regarding whether iodine could be made radioactive had been asked by Dr. Hertz. **b** Response from Dr. Hertz to Dr. Compton dated December 23, 1936. In this response, Dr. Hertz notes that radioactive iodine “will be a useful method of therapy in cases of the overactive thyroid gland”

During the luncheon, Saul Hertz who was in attendance spontaneously asked the very pertinent question that perhaps changed the course of treatment of thyroid disease, “Could iodine be made radioactive artificially?” Saul Hertz was born in 1905 in Cleveland, Ohio. He received his undergraduate degree from the University of Michigan and received his MD degree from Harvard Medical School in 1929. After 2 years of post-graduate training at Mount Sinai Hospital in Cleveland, he returned to Boston in 1931, where he was appointed chief of the thyroid clinic at the MGH. The conversation between Drs. Compton and Hertz can be confirmed by an exchange of letters in the weeks following the luncheon (Fig. 1). It is clear from the letter of Compton that the conversation about the possibility of radioactive iodine was between Drs. Compton and Hertz.

This exchange between Hertz and Compton depended on more than 100 years of prior scientific advances in a number of disparate scientific fields. In 1811, the French chemist Bernard Courtois extracted iodine from the ashes of seaweed. Joseph Louis Gay-Lussac recognized the vapor from the Courtois experiment as a new element and named it “iodine” given its violet color. In 1851, Chatin demonstrated that endemic goiter was associated with low levels of iodine in the drinking water. Kocher significantly improved the surgical approaches to treating thyroid disease and, in 1909, won the Nobel Prize in Medicine and Physiology for his work on the physiology, pathology, and surgery of the thyroid gland. In 1895, Eugen Bauman demonstrated that the thyroid contains iodoprotein, which he called “thyroiodine”, that contained 10–15% iodine and that administration of thyroiodine could reverse myxedema. In 1911, David Marine utilized iodine repletion to treat endemic goiter and treated hyperthyroidism with iodine. In 1922, Means investigates the use of X-rays to treat toxic goiter [3].

In 1896, Henri Bequerel demonstrates that the uranium salts with which he was working emitted radiation similar to the “new kind of rays” that Röntgen had produced with the use of a cathode ray tube just months earlier. Working in Bequerel’s laboratory, Pierre Curie and Marie Sklodowska Curie isolated the naturally occurring radioactive elements polonium and radium. For these accomplishments, Bequerel and Pierre and Marie Curie shared the 1903 Nobel Prize in Physics. In 1923, George de Hevesy demonstrates the tracer principle through his work using radioactive indicators to investigate the absorption and translocation of lead in plants for which he won the Nobel Prize in 1943 [4]. In 1934, Frederic and Iréne Joliot Curie demonstrate the production of artificial radioactivity through their experiments of irradiation of lower Z elements with alpha particles [2]. After alpha irradiation of aluminum, the resultant positrons are emitted, not instantaneously, but with a 2.5 min half-life. They recognize that this phenomenon demonstrates the presence of a new radioactive entity (phosphorus-30). For this accomplishment, Frederic and Iréne Joliot Curie were awarded the 1935 Nobel Prize in Chemistry. Iréne Joliot Curie was the daughter of Marie Curie leading to the only mother–daughter and the 2nd husband–wife (after her parents) recipients of the Nobel Prize.

This collection of scientific accomplishments over the span of the previous 100 years set the stage for Dr. Hertz’s question and the subsequent developments that led to treating the first patient with radioiodine. There are several notable points in the exchange between Drs. Compton and Hertz. Dr. Compton states that iodine can be made artificially radioactive with a half-life of 25 min. Thus, he is referring to ^128^I, which, at that time, could be produced by neutron activation of ^127^I, which had been demonstrated by Enrico Fermi. Dr. Hertz states in his reply to Compton that radioactive iodine may be “a useful method of therapy in cases of overactivity of the thyroid gland.” This extraordinary hypothesis may have been built upon the earlier work by Means regarding the potential treatment of thyroid disease using external X-rays [3].

**Fig. 2:**
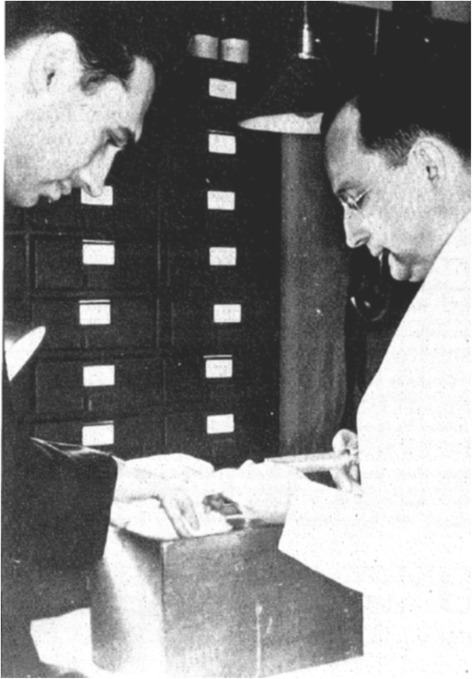
Arthur Roberts (*left*) and Saul Hertz (*right*) performing biokinetic studies of radioiodine in rabbits. The results of these studies were published in 1938 [5]

In the coming years, Dr. Hertz partnered with Arthur Roberts, a young faculty member of the Physics Department of MIT. Their initial work was performed with I^128^ produced using a radium-beryllium (Ra-Be) neutron source to irradiate ^127^I at the MIT Radioactivity Center, which was under the direction of Robley Evans. Utilizing ^128^I, Hertz and Roberts were able to perform the initial biokinetic studies of iodine in rabbits (Fig. 2) and were able to published their findings in 1938 [5]. Although Robley Evans directed the laboratory that provided the radioiodine, the biodistribution studies were performed and analyzed solely by Drs. Hertz and Roberts. At the same time, a group at the University of California in Berkeley led by Joseph Hamilton was performing biodistribution studies of radiosodium and radioiodine [6, 7].

It soon became clear from these biokinetic studies that a radioiodine isotope with a 25-min half-life and the amounts of radioiodine that could be produced with the Ra-Be source would not be optimal for therapeutic applications. 10 years earlier, Ernest Lawrence and his team in Berkeley had developed the cyclotron for various investigations in nuclear physics. After the Joliot Curie paper, it became obvious that the cyclotron also could be used for generating artificial radioactivity. Drs. Compton, Evans, and Means petitioned the Markle Foundation in New York to help fund the installation at MIT of a cyclotron to be used primarily for medical purposes. As a result, the Markle Foundation provided a $30,000 grant for the purchase and installation of the MIT cyclotron. The cyclotron became operational in November 1940 and could produce therapeutic amounts of a mixture of ^130^I (90%) and ^131^I (10%). The half-lives of ^130^I and ^131^I are 12.4 h and 8.1 days, respectively.

**Fig. 3:**
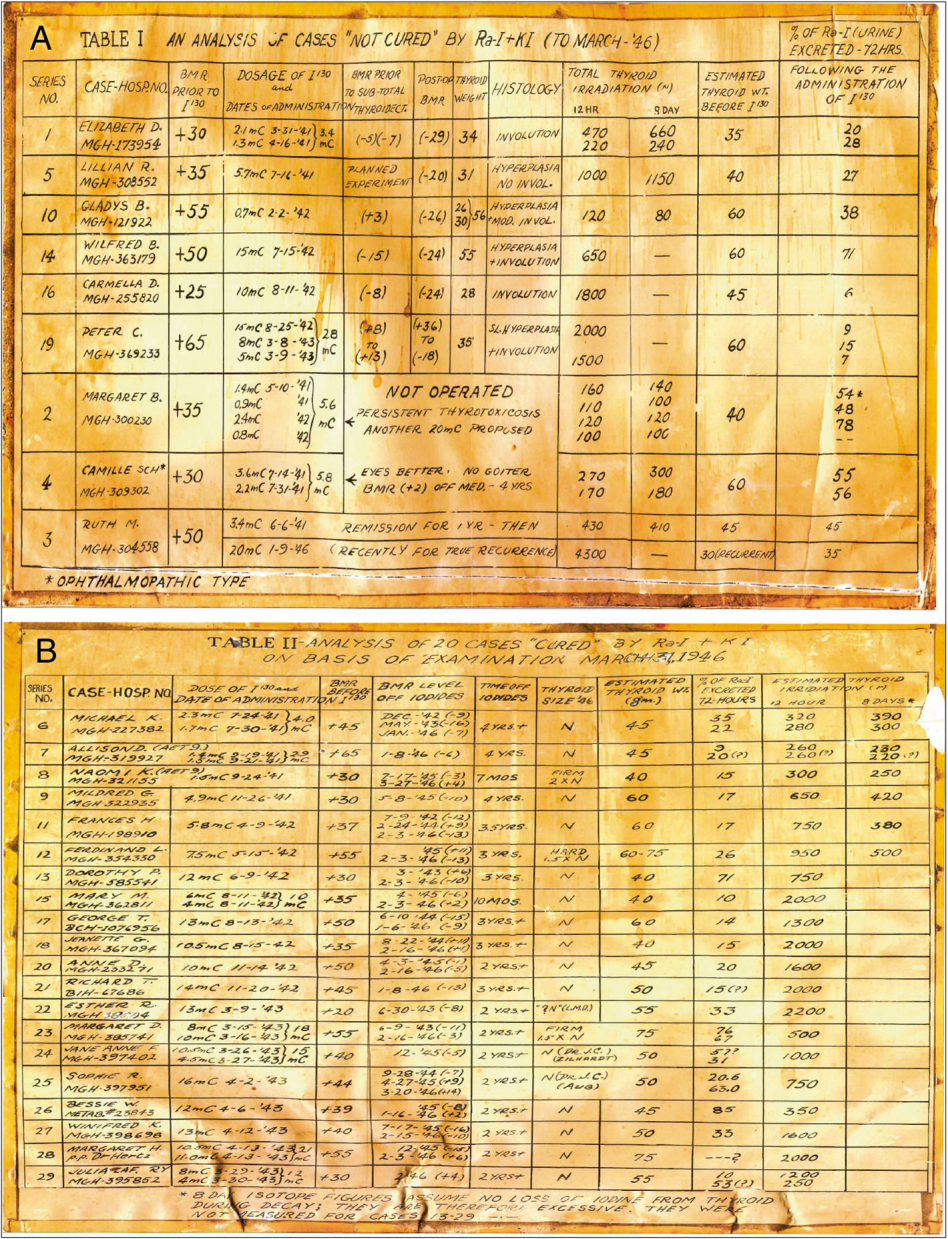
Reproductions of pages from Dr. Hertz’s laboratory notebook showing the result of his first 29 patients. Table 1 (Fig. 3 **a**) lists 9 patients that were "not cured" and subsequently received surgery including ED, the first patient treated with radioiodine for thyroid disease in 1941.  Table 2 (Fig. 3 **b**) lists 20 patients that were cured and required no further treatment

ED, a female patient, is referred to Dr. Hertz’s clinic with hyperthyroidism with no ophthalmopathy and a basal metabolic rate (BMR) of +30. On March 31, 1941, 2.1 mCi of the ^130^I/^131^I mixture is administered to ED, thus she becomes the first patient with thyroid disease to be treated with radioiodine. Upon the advice of Dr. Means, the radioactive treatment is “chased’ with a dose of non-radioactive iodine. 17 days later, ED is treated with an additional 1.3 mCi of radioiodine. After therapy, her BMR decreased to −7. This initial experience was reported in May 1941 at the annual meeting of the American Society for Clinical Investigation. Over the next 2 years, 1941–1943, Hertz and Roberts treat 29 patients and administer post-therapy non-radioactive iodine to all of them. As they later reported [8], 20 of these patients were considered to have been cured and 9 were not cured. The nine patients without resolution of hyperthyroidism underwent thyroidectomy, and upon pathologic examination of the thyroid glands, six had undergone some involution. There were no strong predictors of clinical response. The spreadsheets maintained by Dr. Hertz of these 29 patients are shown in Fig. 3.

In 1943, in the midst of World War II, Dr. Hertz decided to volunteer for military service. Upon his departure, responsibility for the MGH thyroid clinic was turned over to Earle Chapman, MD, who had been working in the clinic under the supervision of Dr. Hertz. While Dr. Hertz was absent, Dr. Chapman worked with Evans to continue to treat thyroid patients with radioiodine. However, in these cases, the administration of radioiodine was not followed with a dose of non-radioactive iodine.

After the war, Dr. Hertz worked to re-establish himself in the medical community in Boston. After being unsuccessful in his attempt to rejoin the staff at the MGH, he joined the medical staff of the Beth Israel Hospital. During this time, it came to Dr. Hertz’s attention that Chapman and Evans planned to publish the results in 22 patients they had treated with radioiodine during the time Hertz was in the service, and that a manuscript without recognition of the work by Hertz and Roberts had been submitted to the Journal of the American Medical Association. Hertz and Roberts reported on their initial 29 patients in the same issue of the journal, just previous to the Chapman and Evans paper. This resulted in the unusual circumstance of two manuscripts on the same topic from the same institution with no overlapping authorship being published back-to-back in the same medical journal [8, 9]. Later the same year, Dr. Seidlin and his group at Montefiore Hospital in New York also reported in the Journal of the American Medical Association on the use of radioiodine to treat 23 patients with metastatic thyroid cancer [10]. Dr. Seidlin had consulted with Dr. Hertz regarding this research. In 1948, Means published a review article on the use of radioiodine in the diagnosis and treatment of thyroid disease [11]. In the article, Means recognized the initial treatment with radioiodine of patients with Graves’ disease by Hertz and Roberts as reported at the American Society for Clinical Investigation in 1941. In this article, he also recognized the subsequent work in this realm by Hamilton and Lawrence as well as Chapman and Evans.

## Conclusions

We have researched these events to the best of our ability, given that they happened over 70 years ago, but we are confident that we have reported the proper sequencing of events so essential to the history of nuclear medicine. Not only do these events mark the first therapeutic use of radioiodine, but also the use of targeted radionuclide therapy to demonstrate the fundamental principles of both molecular nuclear medicine and theranostics. 75 years later, we find ourselves in an era of rebirth for radionuclide therapy, with the recent introductions of ^223^Ra dichloride for treatment of bone metastases and^177^Lu-labeled agents for treatment of both neuroendocrine tumors and prostate cancer. The use of ^177^Lu-labeled agents opens up the potential for new “theranostic” applications, where, much as with radioactive iodine in the management of thyroid disease, the same agent can be used for both diagnostic imaging and therapy. The field of nuclear medicine is indebted to Dr. Hertz, his collaborators, and other early pioneers for their scientific vision, courage, and persistence.
